# Elucidating Film Loss and the Role of Hydrogen Bonding of Adsorbed Redox Enzymes by Electrochemical Quartz Crystal Microbalance Analysis

**DOI:** 10.1021/acscatal.1c04317

**Published:** 2022-01-20

**Authors:** Vivek M. Badiani, Samuel J. Cobb, Andreas Wagner, Ana Rita Oliveira, Sónia Zacarias, Inês A. C. Pereira, Erwin Reisner

**Affiliations:** † Yusuf Hamied Department of Chemistry, University of Cambridge, Lensfield Road, Cambridge CB2 1EW, U.K.; ‡ Cambridge Graphene Centre, University of Cambridge, Cambridge CB3 0FA, U.K.; § Instituto de Tecnologia Química e Biológica António Xavier (ITQB NOVA), Universidade NOVA de Lisboa, Av. da República, 2780-157 Oeiras, Portugal

**Keywords:** hydrogenase, formate dehydrogenase, enzyme immobilization, self-assembled monolayers, bioelectrocatalysis

## Abstract

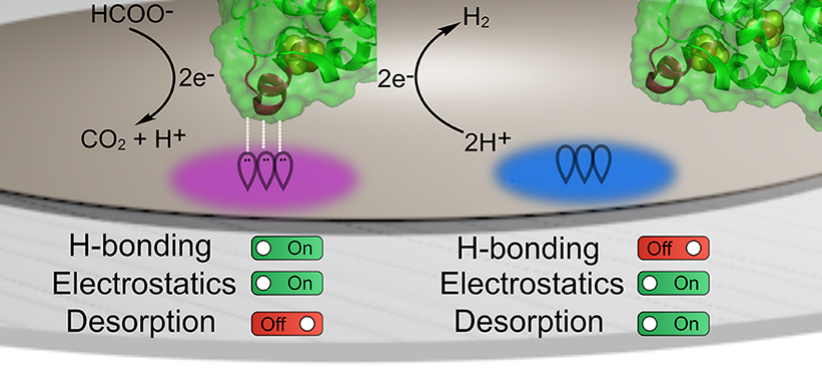

The immobilization of redox enzymes on electrodes enables the efficient and selective electrocatalysis of useful reactions such as the reversible interconversion of dihydrogen (H_2_) to protons (H^+^) and formate to carbon dioxide (CO_2_) with hydrogenase (H_2_ase) and formate dehydrogenase (FDH), respectively. However, their immobilization on electrodes to produce electroactive protein films for direct electron transfer (DET) at the protein–electrode interface is not well understood, and the reasons for their activity loss remain vague, limiting their performance often to hour timescales. Here, we report the immobilization of [NiFeSe]-H_2_ase and [W]-FDH from *Desulfovibrio vulgaris* Hildenborough on a range of charged and neutral self-assembled monolayer (SAM)-modified gold electrodes with varying hydrogen bond (H-bond) donor capabilities. The key factors dominating the activity and stability of the immobilized enzymes are determined using protein film voltammetry (PFV), chronoamperometry (CA), and electrochemical quartz crystal microbalance (E-QCM) analysis. Electrostatic and H-bonding interactions are resolved, with electrostatic interactions responsible for enzyme orientation while enzyme desorption is strongly limited when H-bonding is present at the enzyme–electrode interface. Conversely, enzyme stability is drastically reduced in the absence of H-bonding, and desorptive enzyme loss is confirmed as the main reason for activity decay by E-QCM during CA. This study provides insights into the possible reasons for the reduced activity of immobilized redox enzymes and the role of film loss, particularly H-bonding, in stabilizing bioelectrode performance, promoting avenues for future improvements in bioelectrocatalysis.

## Introduction

Redox enzymes carry out key reactions in biology with unparalleled efficiency at their active sites upon electron exchange with their physiological partners.^1−3^ Enzymes are often large and complex structures, containing transition-metal active sites buried deep within the protein that must be electronically connected to the outer surface via electron transfer centers such as iron–sulfur clusters (FeS).^4^ Charge exchange with the active site is facilitated when the outermost (distal) electron transfer site closely approaches the redox site of the natural redox partner (<14 Å).^5^

The charge flowing to and from a redox enzyme can be redirected in vitro by the immobilization of the isolated enzyme to an electrode.^6^ The stable binding of enzymes to electrode surfaces in an electroactive orientation represents a challenge in fundamental and applied bioelectrochemistry, particularly to elucidate enzyme mechanisms and for their use in sensing as well as catalysis.^7−12^ Orientation control of enzymes on surfaces is critical as it allows the distal electron transfer site to closely approach the electrode for direct electron transfer (DET) in the absence of any redox mediators.

The elucidation of the true turnover frequency (TOF) of enzymes immobilized on electrodes is of key interest; however, disparities in these values exist depending on the technique used to quantify the amount of adsorbed enzymes on the electrode. One method is to analyze the non-turnover signals of the redox centers in the protein, which are observed in the absence of substrate or by inhibition of the enzyme to prevent catalysis from occurring.^7^ This provides an estimate of the amount of electroactive enzymes loaded on the electrode surface. TOFs calculated by this method reach and—in some cases—exceed those measured in solution assays; however, the signal intensities are usually very weak due to the low surface coverage of enzymes (2–5 pmol cm^–2^) and the resulting low density of redox centers, making this technique unreliable for the general use of large proteins such as H_2_ase and FDH.^13^ On the other hand, the gross loading of enzymes can be measured by gravimetric techniques such as quartz crystal microbalance (QCM) and surface plasmon resonance (SPR); however, TOF values are typically on the order of 10^1^ s^–1^, significantly below those determined by solution assays (10^4^ s^–1^), and the reasons for these observed differences are yet to be understood.^14^ The possible explanations include an inaccurate measure of electroactive enzyme loading, a large proportion of enzyme unable to undergo DET due to poor orientation, a considerable amount of immobilized enzymes being denatured and therefore inactive, or a suboptimal environment when the enzyme is immobilized, all of which could be reducing the TOF when compared to freely diffusing in solution.^15−18^

The stability of enzymes on an electrode is another consideration, as ideally the correctly orientated enzyme should maintain activity on the electrode over long periods of time. However, enzymes are susceptible to a range of inactivation mechanisms that may occur during catalysis such as desorption, reorientation, and protein unfolding.^19,20^ Due to the complexity of enzymes and their attachment to surfaces, it is difficult to pinpoint the exact reasons for activity loss, but they can broadly be grouped into non-desorptive (denaturation and reorientation) and desorptive (loss of electroactive enzyme from the electrode) processes. To limit desorptive activity loss, the covalent coupling of the enzyme to the electrode has been used to prevent enzyme leeching from the electrode.^10,21−24^ However, this is chemically non-trivial, and while some reports claimed improvements in long-term activity, others have observed reduced operational stability when compared to a non-covalently bound enzyme.^25^

**Figure 1:**
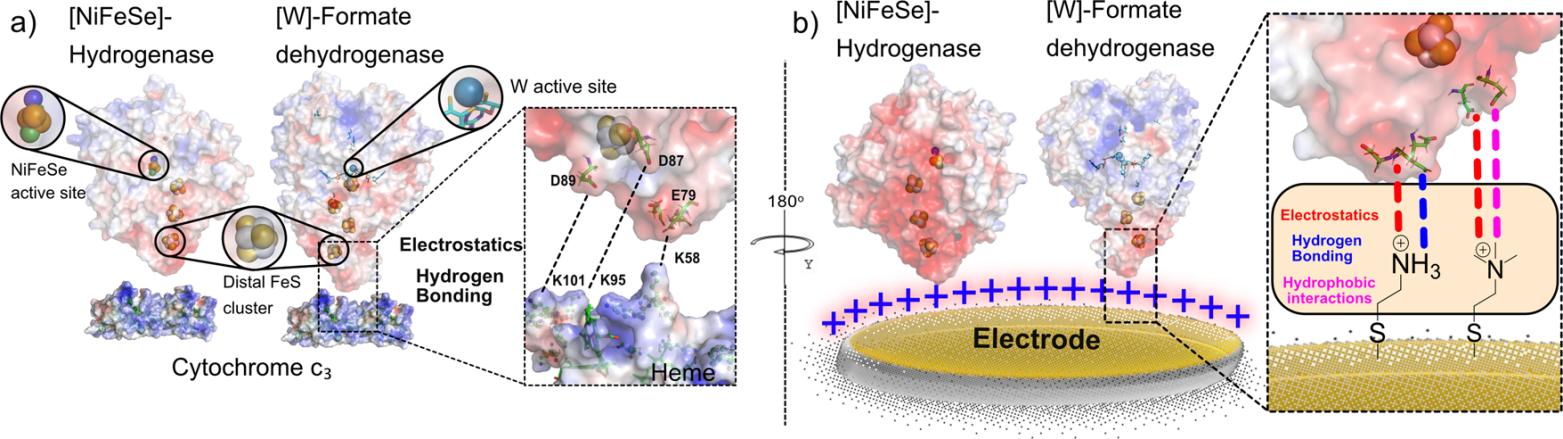
(a) Electrostatic surface potentials (red = negative, blue = positive) across *Dv*H [NiFeSe]-H_2_ase, *Dv*H [W]-FDH, and *Dv*H cyt-*c*_3_ (pdb: 5JSH, 6SDR, and 2CTH, respectively), containing all redox centers including hemes, FeS clusters, the NiFeSe and W active sites, molybdopterin cofactors, and their in vivo electrostatic and hydrogen bonding interactions to one another (inset). (b) Oriented immobilization of *Dv*H [NiFeSe]-H_2_ase and [W]-FDH on a SAM-modified Au electrode with hydrogen bonding and non-hydrogen bonding SAMs and the possible non-covalent interactions at the enzyme–electrode interface (inset).

Therefore, to improve the DET performance and stable integration in bioelectrodes, the enzyme–electrode interface must be understood and can be designed to provide a desirable interaction by taking inspiration from natural biological enzyme–redox partner interactions.^18^ [NiFeSe]-hydrogenase (H_2_ase)^26^ and [W]-formate dehydrogenase (FDH)^27^ from *Desulfovibrio vulgaris* Hildenborough (*Dv*H) are examples of highly efficient redox enzymes that can reversibly interconvert protons (H^+^)/H_2_ and CO_2_/HCO_2_^–^, respectively.^28,29^ Although they are different enzymes with distinct protein structures and active sites, they both possess a natural dipole moment with a local negative region around the distal FeS cluster. This interacts strongly with a local positive region near the electron-accepting heme of the natural redox partner cytochrome *c*_3_ (cyt-*c*_3_)^1,2^ to enable fast and efficient electron transfer in vivo (Figure 1a). This can provide inspiration for the design of a chemically modified electrode surface that can orientate the enzyme in the same manner as the cyt-*c*_3_, as mimicking the enzyme’s natural environment on an electrode offers the best opportunity to match the high activities achieved in vivo.

Electrode materials for enzyme immobilization can range from carbon (graphite, carbon nanotubes, graphene) to metal oxides (indium tin oxide (ITO), titanium dioxide (TiO_2_)), and gold (Au).^30^ Carbon electrodes, such as pyrolytic graphite edge and carbon nanotubes, have been successfully used to immobilize redox enzymes for DET, but they contain different aromatic, hydroxyl, and carbonyl moieties and are non-planar, complicating the ability to control and study the orientation of a protein.^10,18,31,32^ Metal oxides such as ITO and TiO_2_ have also been used for the immobilization of H_2_ase and FDH and the resulting protein film exploited in electrocatalysis and solar fuel synthesis.^9,33−37^ However, while these materials are desirable for many applications, their surface terminations are often not well defined and it is also challenging to engineer their surfaces to mimic the enzyme’s natural redox partner. While ITO and TiO_2_ colloids can be chemically modified with alkylphosphonic acids, they are prone to hydrolyze in aqueous solutions, display instabilities (ITO) or a lack of conductivity (TiO_2_) at certain potential ranges,^38^ and can yield disordered self-assembled monolayers (SAMs) due to the inefficient packing on the rough metal oxide surface.^39^

Au is a highly planar noble metal that is stable over a wide redox window and can be easily modified with thiols to form stable, highly-ordered SAMs that have been thoroughly characterized, providing a well-defined, surface-tunable model electrode surface on which to immobilize redox enzymes.^40,41^ The SAM can be designed to control the enzyme orientation and be exploited to probe the effects of surface termination on enzyme stability (Figure 1b).^41−43^ Moreover, the use of Au enables spectro-electrochemical approaches, such as surface-enhanced infrared absorption spectroscopy, as well as gravimetric techniques such as QCM.^14,44^ This allows for the operando study of the vibrational structure of the protein backbone and the active site of redox enzymes, as well as their adsorption onto surfaces, making this the ideal electrode to understand the enzyme–electrode interface,^45,46^ providing information that can then be transferred to other less well-defined surfaces such as carbon and metal oxides to improve the performance of enzymes for applications such as biofuel cells and photoelectrochemical devices.^35−37,47,48^

The oriented immobilization of H_2_ase^22,49−52^ and FDH^17,23,53,54^ for DET on electrodes has been demonstrated, but the reasons for their activity loss are not fully understood and are commonly described as “film loss” to encompass a varied range of processes speculated to be responsible for observed decreases in current density.^15,47^

To understand the activity and stability of H_2_ase and FDH on electrodes, the exact nature of the binding of physisorbed redox enzymes on modified electrodes needs to be understood as rational design is one of the most promising means to provide the step change in activities necessary to allow enzymes to approach their maximum activities determined in solution.^55^ For example, protein binding to the modified electrodes is often assumed to be mainly governed by electrostatic interactions, yet additional non-covalent interactions such as hydrogen bonding (H-bonding), hydrophobic interactions, and van der Waals (vdW) interactions, which can exist in vivo with their respective redox partners, are less commonly investigated, and their net contribution to bioelectrocatalytic performance is not well known.^53,56^ Moreover, these interactions may be important for the effective immobilization of enzymes on surfaces and could provide a greater understanding of the nature and contribution of the multiple interactions present that are required to develop systems with better enzyme orientation, activity, and stability.

In this work, the non-covalent interactions that govern enzyme orientation, binding, activity, and stability at the enzyme–electrode interface were elucidated for *Dv*H H_2_ase and FDH. The influence of electrostatics and H-bonding on enzyme immobilization and orientation was probed on a range of SAM–Au electrodes using protein film voltammetry (PFV), chronoamperometry (CA), and electrochemical quartz crystal microbalance (E-QCM) analysis. Using rationally chosen SAMs, strong evidence for the presence and role of H-bonding interactions in protein stabilization, similar to those thought to exist in vivo, was observed at the enzyme–electrode interface, providing an insight into the approaches needed to improve redox enzyme performance on electrodes to allow them to approach their maximal rates.^25,57^

## Results and Discussion

### Electrostatic Orientation on SAM-Modified Au Electrodes

**Figure 2:**
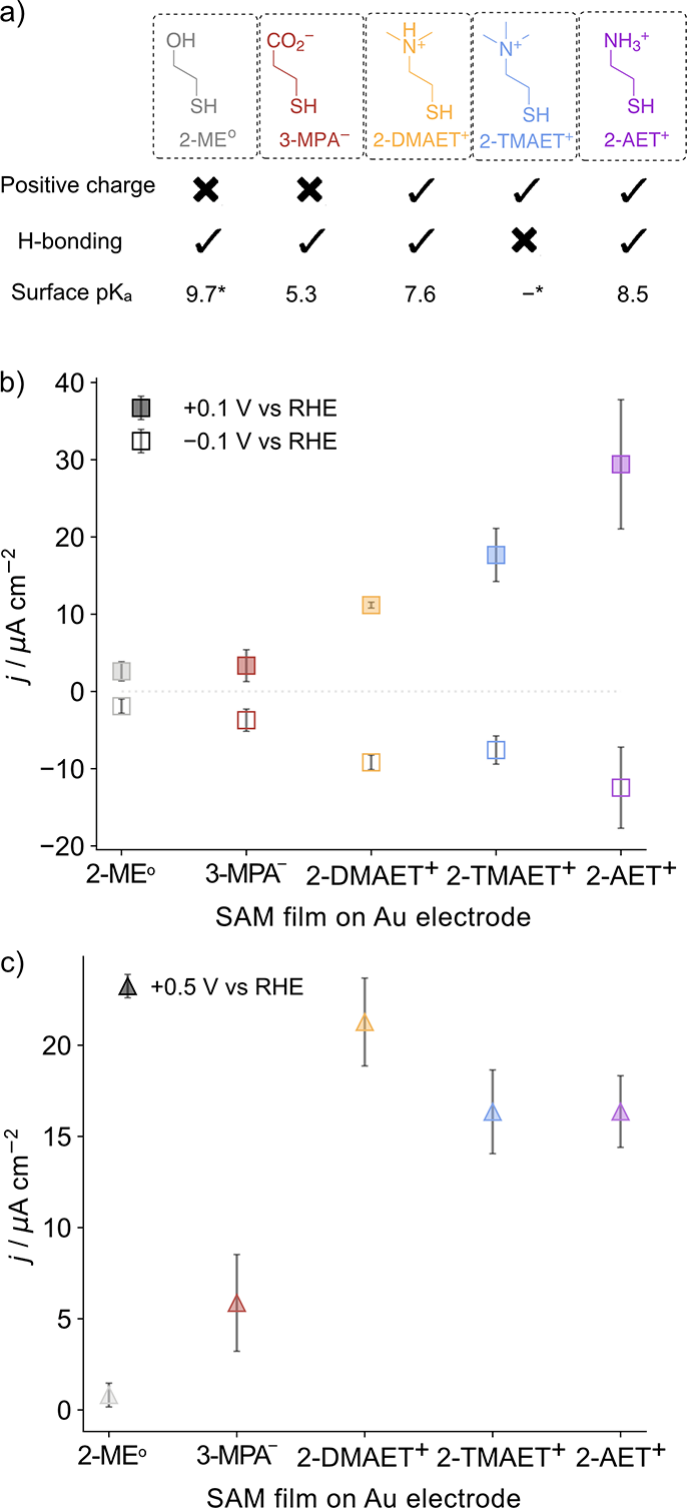
(a) Thiol-based SAMs used to control the enzyme orientation and stability on the electrode, their electrostatic and H-bonding properties at pH 6, and their surface p*K*_a_ values. The asterisk denotes the bulk solution p*K*_a_ in the case where the surface p*K*_a_ value is not available (2-ME^o^) or where a pH-independent head group is employed (2-TMAET^+^). (b) Activity of H_2_ase adsorbed on different SAM-modified gold electrodes taken from the current recorded by PFV at +0.1 V (filled squares) and −0.1 V vs RHE (empty squares). (c) Activity of FDH adsorbed on different SAM-modified gold electrodes taken from the current recorded by PFV at +0.5 V vs RHE (filled triangles). Conditions: MES/KCl (50 mM/50 mM, pH 6), 1 atm H_2_ for H_2_ase (10 pmol), and HEPES/KCl/formate (50 mM/50 mM/20 mM, pH 8) for FDH (40 pmol) activated by incubation with 1,4-dithiothreitol (DTT, 50 mM). ν = 5 mV s^–1^, ω = 2000 rpm, 25 °C. Error bars represent the standard deviation for a sample size of *n* = 3.

First, SAM-modified Au electrodes were prepared by immersing a gold rotating disk electrode (RDE) (geometric area = 0.0314 cm^2^) in aqueous solutions of the relevant thiol (10 mM) overnight. Five SAMs were used to represent different charges and H-bonding abilities: 2-mercaptoethanol (2-ME^o^), 3-mercaptopropionate (3-MPA^–^), 2-dimethylammoniumethanethiol (2-DMAET^+^), 2-trimethylammoniumethanethiol (2-TMAET^+^), and 2-ammoniumethanethiol (2-AET^+^) (Figure 2a). At pH 6, according to their respective p*K*_a_ (Figure 2a), these thiols generate a surface charge denoted by their superscript.^21,58−62^ To confirm the assembly and net charge of thiol-based SAMs on gold, 2-AET^+^ was functionalized onto gold nanoparticles, and the pH-dependent charge was confirmed by zeta-potential measurements (Figure S1).

The orientation of H_2_ase and FDH immobilized on the SAM-modified electrodes at pH 6 was found to be strongly dependent on the surface charge. Good’s buffers at pH 6 for H_2_ase and pH 8 for FDH were selected to provide optimal electrolyte conditions for proton reduction^26^ and formate oxidation,^27^ respectively—the reactions of interest in the subsequent E-QCM experiments. Bubble formation from CO_2_-purged buffers in the E-QCM experiments prevented the accurate study of CO_2_ reduction with FDH.

Electrochemical DET activity for both H^+^ reduction and H_2_ oxidation for H_2_ase and formate oxidation for FDH was highest using the positively charged SAMs 2-DMAET^+^, 2-TMAET^+^, and 2-AET^+^, which can be ascribed to the electrostatic attraction to the negatively charged region surrounding the distal FeS cluster in both enzymes (Figure 2b,c).

The electrocatalytic waveshape of FDH immobilized on the three positively charged electrodes (2-AET^+^, 2-DMAET^+^, 2-TMAET^+^) displayed hysteresis under N_2_ (Figure S2b), which disappeared upon saturation of the electrolyte with CO_2_ with a concurrent decrease in activity (Figure S3). This is likely due to the oxidation of formate to CO_2_ at positive overpotentials in the N_2_-saturated electrolyte, which affects the intrinsic activity of the enzyme on the reverse scan possibly due to the saturation of the substrate channels of the enzyme, leading to hysteresis. A similar inhibition of H_2_ase was observed in the presence of H_2_.^19^ To retain sufficient activity and to prevent the use of CO_2_-purged buffers, which would affect subsequent E-QCM experiments, an N_2_-saturated electrolyte was used for all FDH experiments, and a potential of +0.5 V vs RHE was chosen for current analysis, which is the point at which hysteresis is at a minimum, allowing for confidence in the currents analyzed.

**Figure 3:**
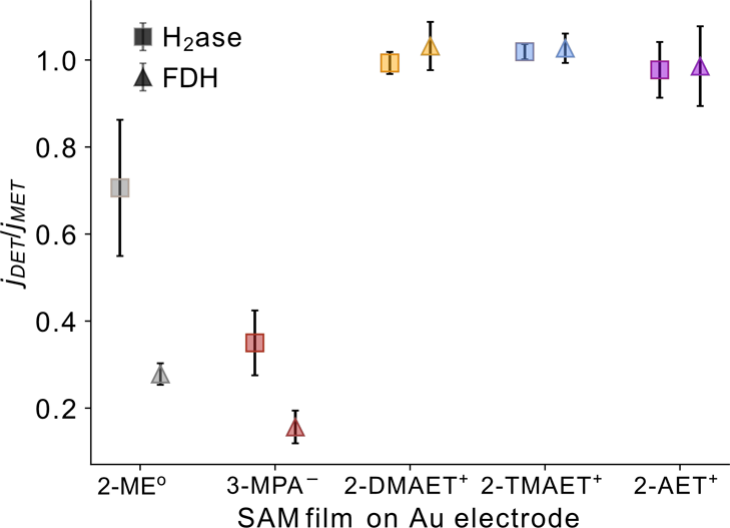
DET to MET current density ratio extracted from the PFV response for H_2_ase for H_2_ oxidation at +0.1 V vs RHE (squares) and FDH for formate oxidation at +0.5 V vs RHE (triangles) on each SAM-modified electrode. Conditions: H_2_ase (10 pmol), MES/KCl (50 mM/50 mM, pH 6), 1 atm H_2_, MV^2+^ (250 μM). FDH (40 pmol), DTT (50 mM), HEPES/KCl/formate (50 mM/50 mM/20 mM, pH 8), BV^2+^ (250 μM). ν = 5 mV s^–1^, ω = 2000 rpm, 25 °C. Error bars represent the standard deviation for a sample size of *n* = 3.

The redox mediators methyl viologen (MV^2+^, 250 μM, *E*^0^′ = −0.09 V vs RHE at pH 6) and benzyl viologen (BV^2+^, 250 μM, *E*^0^′ = +0.11 V vs RHE at pH 8) can be used to estimate the amount of enzyme immobilized on the surface irrespective of orientation via mediated electron transfer (MET) as any active enzyme not oriented via the distal FeS cluster can undergo MET, resulting in an increase in current density (*j*).^18,63^ Subsequently, the amount of the DET current compared to the MET current provides a *j*_DET_/*j*_MET_ value, which signifies the proportion of the enzyme bound to the electrode in a favorable orientation via the distal FeS cluster, where *j*_MET_ includes any contribution from DET. Adding mediators showed little to no net increase over the DET currents for H_2_ oxidation by H_2_ase or formate oxidation by FDH on the three positively charged electrodes, which suggests near quantitative binding of H_2_ase and FDH in the correct orientation for DET as evidenced by a near unity *j*_DET_/*j*_MET_ value (Figure 3).

In contrast, a lower *j* was observed for 2-ME^o^, which indicates non-optimal orientation, and only a fraction of the enzyme immobilized in the DET orientation (Figure 2b,c). The addition of redox mediators led to an increase in anodic *j* (*j*_ox_) for both enzymes on the neutral electrode (Figure 3), although this was less pronounced for H_2_ase, as shown by the catalytic waveshape in the representative protein film voltammograms (Figure S2).^18^

A similarly low *j*_ox_ of 3.3 ± 2 and 5.9 ± 2.7 μA cm^–2^ was observed for H_2_ase and FDH on 3-MPA^–^, respectively, which can be assigned to the electrostatic repulsion of the distal FeS region of the enzymes (Figure 2b,c). The poor orientation of the enzymes on 2-ME^o^ and 3-MPA^–^ was confirmed by the addition of redox mediators, which significantly increased *j*_ox_, resulting in a *j*_DET_/*j*_MET_ of 0.35 ± 0.07 and 0.16 ± 0.04 for H_2_ase and FDH, respectively on 3-MPA^–^ (Figure 3). An enzyme-free CV in the presence of BV^2+^ displayed a much lower *j*_ox_ than in the presence of FDH, confirming that the increase in *j*_ox_ was due to enzymatic MET (Figure S4).

### Stability of the Enzyme Film by CA

**Figure 4:**
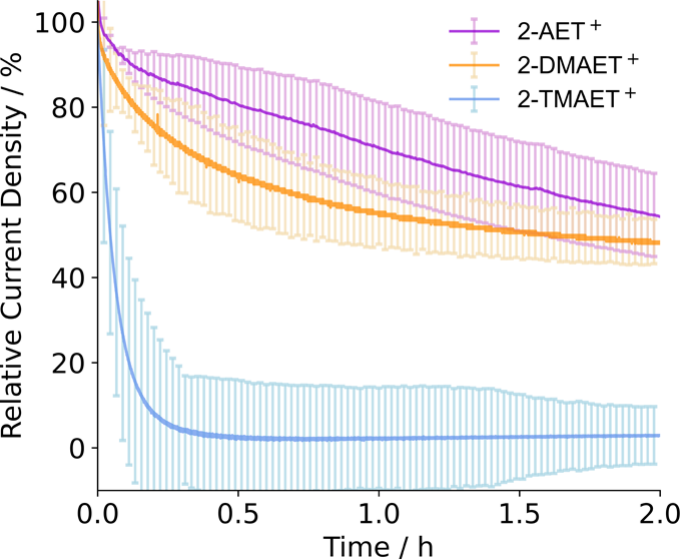
CA of H_2_ase for H_2_ oxidation adsorbed on 2-AET^+^, 2-DMAET^+^ and 2-TMAET^+^. Conditions: MES/KCl (50 mM/50 mM, pH 6), 1 atm H_2_, H_2_ase (10 pmol), 25 °C, ω = 2000 rpm, *E*_app_ = +0.1 V vs RHE. Error bars represent the standard deviation for a sample size of *n* = 3.

In addition to orientation in an electroactive configuration, the design of electrode surfaces to promote enzyme films with high stability on electrodes is another key feature in bioelectrocatalysis. The electrocatalytic stability of H_2_ase on each of the well-orientated positively charged electrodes was therefore assessed by CA at an applied potential (*E*_app_) of +0.1 V vs RHE (Figure 4). 2-AET^+^ and 2-DMAET^+^ displayed a similar decrease in electrocatalytic activity, with a loss in current of (46 ± 9.6) and (52 ± 5.4)% over 2 h, supporting that the film stability was not detrimentally affected by the presence of methyl moieties. On the other hand, 2-TMAET^+^ exhibited a rapid current decay in the first 20 min and lost (97 ± 6.7)% of its activity over 2 h. This difference may be explained by the differing H-bonding abilities of each SAM on the electrode. 2-AET^+^ and 2-DMAET^+^ are protonated primary and secondary amines and can act as strong H-bond donors,^64^ whereas 2-TMAET^+^ is a quaternary ammonium cation that cannot act as a H-bond donor.^65^

The reasons for the loss in electrocatalytic activity for each of the H_2_ase-films can be attributed to either desorptive or non-desorptive processes, which cannot be determined by electrochemistry alone. To further understand the factors contributing to film loss, gravimetric techniques such as QCM combined with electrochemistry can provide a better understanding of the enzyme–electrode interface.

### Investigating Electrostatic Interactions by E-QCM

**Figure 5:**
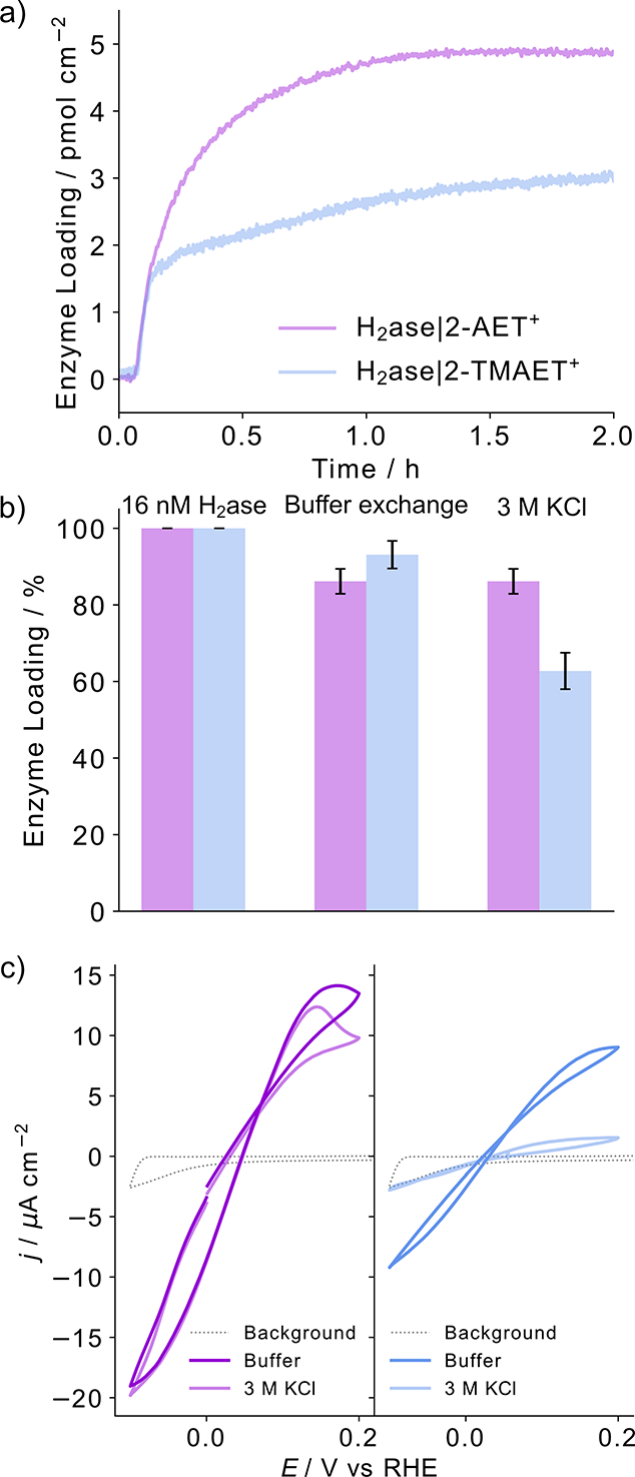
(a) QCM profile for the immobilization of H_2_ase on 2-AET^+^- and 2-TMAET^+^-modified Au sensors. (b) Desorption profiles of H_2_ase on the SAM-modified Au sensors after a buffer exchange followed by exposure to 3 M KCl. (c) PFV responses of H_2_ase on the SAM-modified sensors after buffer exchange and 3 M KCl. Conditions: MES/KCl (50 mM/50 mM, pH 6), 1 atm H_2_, H_2_ase (16 nM), flow rate = 0.141 mL min^–1^, 25 °C. Error bars represent the standard deviation for a sample size of *n* = 3 across three independent sensors.

QCM can quantify the loading of enzymes on an electrode and, in combination with electrochemical analysis (E-QCM), can be used to probe changes at the enzyme–electrode interface under turnover conditions.^44^ A typical monolayer film of H_2_ase on 2-AET^+^-modified gold QCM sensors reached (5.0 ± 0.3) pmol cm^–2^, whereas a lower loading was observed for 2-TMAET^+^ ((3.2 ± 0.1) pmol cm^–2^; Figure 5a; eq 1). These surface coverages are similar to the monolayer coverage observed previously for H_2_ase on planar TiO_2_^37^ and are comparable to a theoretical monolayer loading of 3–10 pmol cm^–2^ of H_2_ase (8.5 nm × 7.5 nm × 6.5 nm) depending on the orientation of the enzyme upon immobilization. Therefore, the loading values generated by E-QCM can be used to accurately determine TOFs without the uncertainty of an assumed enzyme loading provided that the majority of the immobilized enzymes are orientated for DET. However, limitations arise here as the proportion of loaded enzymes that may be denatured upon immobilization is unknown.

The strength of the enzyme–electrode interaction was probed by washing the H_2_ase-adsorbed SAM-modified electrodes with an enzyme-free MES/KCl (50 mM/50 mM, pH 6) buffer, followed by 3 M KCl to shield electrostatic interactions between the enzyme and the electrode and decouple the ratio of electrostatically bound to non-electrostatically bound H_2_ase (Figure 5b). A small decrease in adsorbed H_2_ase was observed for both 2-AET^+^ and 2-TMAET^+^ when switching from the denser enzyme-containing solution to the enzyme-free buffer signaling the removal of physisorbed enzymes bound on top of the underlying monolayer protein film, but surprisingly, no further H_2_ase desorption was observed after 3 M KCl on 2-AET^+^ indicating that all enzyme molecules were bound by non-electrostatic interactions. FDH also remained quantitatively bound to 2-AET^+^ after exposure to 3 M KCl (Figure S5b), confirming that this effect is present across two different enzymes with similar surface charge properties.

This observation is unexpected as the most commonly referenced physisorbed interaction at the enzyme–electrode interface is electrostatic, without in-depth reference to other possible interactions.^56,66^ The activity of the remaining H_2_ase on 2-AET^+^ was confirmed by protein film voltammograms in the MES/KCl (50 mM/50 mM, pH 6) buffer solution after each ionic solution washing step, where a negligible current loss was observed after exposure to 3 M KCl (Figure 5c), with a quantitative activity also observed for FDH on 2-AET^+^ after 3 M KCl (Figure S5c). Additionally, no desorption was observed by QCM for H_2_ase washed with 3 M KCl on 3-MPA^–^ even though the negative charge of the electrode misorients the enzyme, supporting that the protein is still bound strongly to the electrode by other non-covalent interactions regardless of orientation (Figure S6b). Enzyme–enzyme interactions were also investigated by loading only half of the expected monolayer of H_2_ase on 2-AET^+^ followed by the same washing procedure, with no desorption occurring after 3 M KCl (Figure S7). This hints that the enzyme was stable on the surface at sub-monolayer coverages where the possibility of interactions between enzymes may be minimized due to regions of increased inter-enzyme spacing.

This provides strong evidence for distinct non-covalent interactions that separately govern orientation and immobilization at the enzyme–electrode interface. This is also possible for FDH on TiO_2_ where 60% of FDH remained adsorbed after exposure to 3 M KCl.^54^ Due to the presence of H-bonding between H_2_ase and cyt-*c*_3,_^67,68^ it is possible that H-bonding is the main non-covalent interaction playing a role in immobilization at the H_2_ase-2-AET^+^ interface, preventing enzyme desorption when electrostatic interactions are shielded by the 3 M KCl solution. H-bonding for physisorbed enzymes to electrodes has been suggested previously for Cu,Zn superoxide dismutase on cysteine-modified Au electrodes, where H-bonding between cysteine and threonine was proposed, although no evidence for this interaction was provided.^69,70^

To validate the hypothesis of H-bonding, H_2_ase adsorbed on the 2-TMAET^+^-modified QCM Au sensor was subject to 3 M KCl, and a loss of (38 ± 5)% compared to the initial loading of H_2_ase was observed (Figure 5b), while FDH adsorbed on 2-TMAET^+^ led to a loss of (20 ± 8)% compared to the initial loading (Figure S8b). This could be rationalized by the absence of H-bonding between the enzyme and 2-TMAET^+^, leading to a larger contribution from electrostatic interactions to immobilization, ultimately prompting enzyme desorption at high salt concentrations when these interactions were shielded. Mediator-free protein film voltammograms recorded after the observed desorption confirmed the loss of H_2_ase (Figure 5c) and FDH (Figure S8c) from the 2-TMAET^+^ sensor, with the current decreasing by a larger proportion than the loading possibly being due to the non-desorptive activity loss such as reorientation or active site degradation occurring simultaneously with the desorptive activity loss. To confirm that the desorption of protein from 2-TMAET^+^ was not due to a more hydrophobic surface, H_2_ase was loaded onto a propanethiol-modified Au sensor, a purely hydrophobic and non-electrostatic surface, whereupon no desorption after 3 M KCl was observed, indicating that hydrophobic interactions between the enzyme and electrode were also stable in the presence of high ionic concentrations (Figure S9).

Due to the previous observation of a near quantitative amount of enzymes orientated for DET on the positively charged electrodes (Figure 3), TOFs of the monolayer enzyme films were extracted from the loading and the electrocatalytic data by E-QCM (Table 1) with the assumption that *j*_DET_/*j*_MET_ at −0.1 V vs RHE and +0.5 V vs RHE for H_2_ase and FDH, respectively, on 2-AET^+^ and 2-TMAET^+^ is 1 (Figures S10, S11). The highest apparent TOFs (TOF_apparent_) were observed on 2-AET^+^ (21.1 ± 1.8 s^–1^ for H_2_ase and 17.5 ± 0.8 s^–1^ for FDH) and 2-TMAET^+^ (17.7 ± 2.9 s^–1^ for H_2_ase and 26.8 ± 1.2 s^–1^ for FDH) taking into account the gross enzyme loading by QCM. This is much lower than the activities observed by conventional solution assays for H_2_ase and FDH (5201 and 1100 s^–1^ for H^+^ reduction and formate oxidation, respectively, see the Materials section), which was also observed for bilirubin oxidase on SAM-modified Au by electrochemical SPR and E-QCM.^25,71,72^ The reason for this discrepancy is unclear and remains a common challenge in the field of PFV (Table S1).^17,49^

**Table 1:** Summary of Information Obtained from E-QCM Analysis for H_2_ase and FDH on SAM-Modified Au Sensors

enzymatic system^a^	total loading (pmol cm^–2^)^b^	electroactive loading (pmol cm^–2^)^c^	|j_DET_| (μA cm^–2^)^d^	|j_MET_| (μA cm^–2^)^e^	j_DET_/j_MET_	TOF_apparent_ (s^–1^)^f^	TOF_actual_ (s^–1^)^g^
H_2_ase|2-AET^+^|_DET_	5.1 ± 0.1	5.1 ± 0.1	20.6 ± 1.8	20.6 ± 1.8	1.0	21.1 ± 1.8	21.1 ± 1.8
H_2_ase|2-TMAET^+^|_DET_	3.2 ± 0.1	3.2 ± 0.1	10.9 ± 1.4	10.9 ± 1.4	1.0	17.7 ± 2.9	17.7 ± 2.9
FDH|2-AET^+^|_DET_	3.7 ± 0.3	3.7 ± 0.3	12.5 ± 0.5	12.5 ± 0.5	1.0	17.5 ± 0.8	17.5 ± 0.8
FDH|2-TMAET^+^|_DET_	2.8 ± 0.2	2.8 ± 0.2	14.4 ± 0.6	14.4 ± 0.6	1.0	26.8 ± 1.2	26.8 ± 1.2
H_2_ase|3-MPA^–^|_DET_	4.4 ± 0.2	0.26 ± 0.02	1.27 ± 0.1	21.0 ± 1.3	0.06 ± 0.01	1.5 ± 0.1	25.4 ± 1.8
H_2_ase|3-MPA^–^|_MET_	4.4 ± 0.2	4.4 ± 0.2		21.0 ± 1.3			25.9 ± 1.6

a The subscripts _DET_ or _MET_ refers to the current density (*j*_DET_ or *j*_MET_) used when calculating the catalytic current *i* used in eqs 2 and 3.

b Total loading is calculated using eq 1 in the Experimental Section.

c Electroactive loading is calculated by considering *j*_DET_/*j*_MET_ values for each enzymatic system.

d Values of |*j_DET_*| for H_2_ase are obtained from PFV responses at *E* = −0.1 V vs RHE, while |*j_DET_*| values for FDH are obtained from PFV responses at *E* = +0.5 V vs RHE.

e Values of |*j*_MET_| are obtained by the addition of 250 μM MV^2+^ (H_2_ase) or BV^2+^ (FDH).

f TOF_apparent_ is calculated using eq 2 in the Experimental Section.

g TOF_actual_ is calculated using eq 3 in the Experimental Section, taking into account *j*_DET_/*j*_MET,_ where *j*_MET_ includes the contribution from DET. All data were acquired from E-QCM experiments.

In this work, we have so far been able to rule out poor orientation and enzyme desorption as factors that contribute to the low electrochemical TOFs, and although this work signifies progress toward an understanding of why the electrochemical TOFs are much lower than solution TOFs, further work in the PFV field is necessary to elucidate this. Some reasons for this difference in activity could be (i) protein deconformation upon immobilization with a heterogeneous substrate as opposed to a homogeneous soluble redox partner (redox mediator in vitro or cyt-*c*_3_ in vivo), (ii) protein crowding on electrodes altering the rate of enzyme reactions, or (iii) electric field-induced protein denaturation.^15,71,73−76^ One key question that remains is whether the majority of the loaded enzymes are inactive or whether the intrinsic activity of each enzyme molecule is lower, and consequently, the method used to calculate the TOF is extremely important. From our results, the low TOFs on electrodes convey the need to better understand and optimize interfacial electron transfer by methods other than solely orientation to realize limiting currents similar to the activities observed in solution assays.

Nevertheless, the electrode activity values herein are similar to DET TOFs observed for various redox enzymes on SAM–Au, metal oxide, functionalized graphite, Ketjen Black, and carbon cloth electrodes, thus providing a comparable system with the current state of the art in the PFV field with which to further analyze the function of non-covalent interactions at the enzyme–electrode interface (Table S1).^9,71,75,77−79^

H_2_ase loaded on 3-MPA^–^ displayed a significantly lower TOF_apparent_ of 1.5 ± 0.1 s^–1^ by E-QCM, initially indicating that the TOF is limited by electron transfer due to the suboptimal orientation of the enzyme molecules on the electrode. However, the TOF_apparent_ considers only the total enzyme loading (eq 2). Therefore, to calculate the real TOF (TOF_actual_) of H_2_ase on 3-MPA^–^, the actual amount of electroactive enzyme (electroactive loading) generating catalytic current must be considered as opposed to the total amount of immobilized enzymes (eq 3). This was realized by the addition of MV^2+^ to the H_2_ase-adsorbed 3-MPA^–^ QCM sensor, which led to a drastic increase in the current density (|*j*|) from (1.27 ± 0.1) μA cm^–2^ to (21.0 ± 1.3) μA cm^–2^, exhibiting a *j*_DET/_*j*_MET_ of 0.06 ± 0.01 at a total loading of 4.4 ± 0.2 pmol cm^–2^ (Figure S6c). Therefore, assuming that the intrinsic enzyme activity is unaffected by orientation of the total H_2_ase loaded on 3-MPA^–^, only (0.26 ± 0.02) pmol cm^–2^ of H_2_ase are in direct electronic communication with the 3-MPA^–^ electrode. This results in a TOF_actual_ of 25.4 ± 1.8 s^–1^, within the same order of magnitude of H_2_ase on the positively charged electrodes, and within the error of the TOF_actual_ of 25.9 ± 1.6 s^–1^ for H_2_ase on 3-MPA^–^ in the presence of the redox mediator MV^2+^ (Figure S6c), confirming that intrinsic enzyme activity is retained on negative electrodes when compared to positive electrodes but still much lower than their activity in solution. This analysis emphasizes the importance of knowing both the loading and orientation information to accurately measure an enzyme TOF, something which is not sufficiently considered in the bioelectrocatalysis field.

### Deconvoluting Desorptive and Non-desorptive Activity Loss with E-QCM

**Figure 6:**
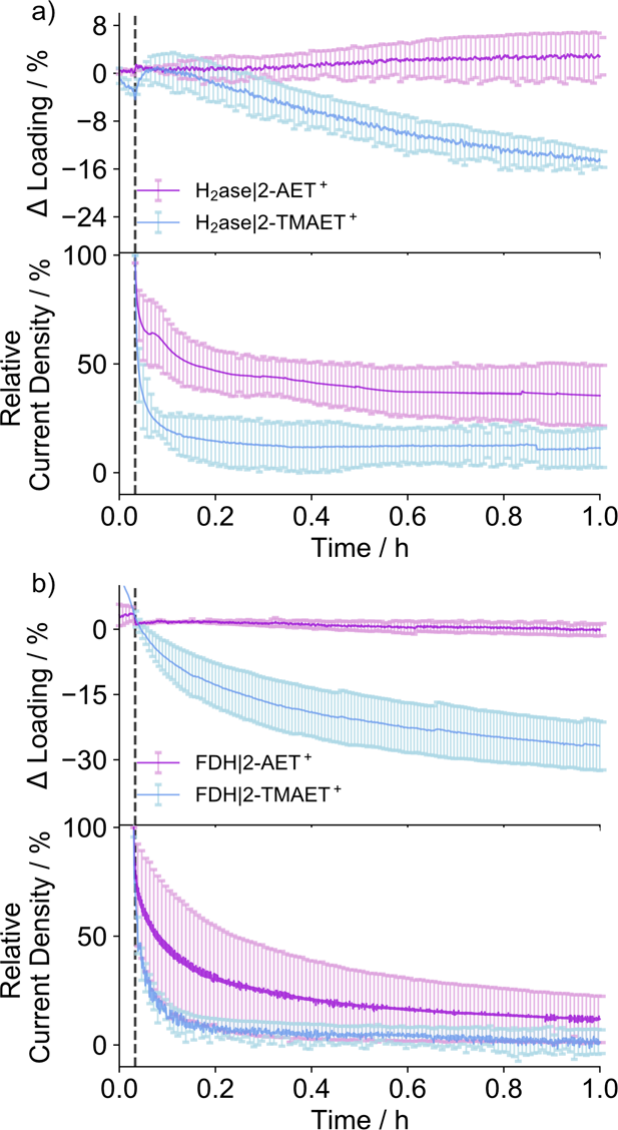
E-QCM CA after loading (a) H_2_ase and (b) FDH on 2-AET^+^ and 2-TMAET^+^ sensors (lower panel) and their corresponding changes in loading (upper panel) operando. The shapes of the loading curves before the vertical dotted line are due to a 2 min pre-equilibration applied at the *E*_app_ to prevent large capacitance spikes at the start of the current measurement. The vertical dotted line indicates the start of the CA measurement at the applied potential, *E*_app_ = −0.1 V vs RHE (H_2_ase, H^+^ reduction) and +0.4 V vs RHE (FDH, formate oxidation) after a 2 min electrode equilibration. Conditions: MES/KCl (50 mM/50 mM, pH 6), H_2_ase (16 nM). HEPES/KCl/formate (50 mM/ 50 mM/20 mM, pH 8), FDH (66 nM), DTT (50 mM), flow rate = 0.141 mL min^–1^, N_2_ atmosphere, 25 °C. Error bars represent the standard deviation for a sample size of *n* = 3.

E-QCM allows for monitoring the change in surface coverage in operando and to probe the different surface conditions leading to either desorptive or non-desorptive activity loss. CA was performed on 2-AET^+^ and 2-TMAET^+^ QCM Au sensors preloaded with 5.0 and 3.2 pmol H_2_ase cm^–2^, respectively, after which a 2 min pre-equilibration was applied at a constant potential before beginning the CA. Figure 6a shows the change in enzyme loading (top) and current decay (bottom) during CA. No change in protein loading was observed over 1 h, but the proton reduction current at −0.1 V vs RHE decays by (65 ± 10)% for the H-bond capable 2-AET^+^, indicating that the mechanism for the activity loss was non-desorptive due to the presence of H-bonds between the protein and the electrode. It was previously speculated that strong electrostatic interactions may destabilize bilirubin oxidase on 6-mercaptohexanoic acid-modified electrodes,^75^ which could be a possible driver of the observed non-desorptive activity loss for H_2_ase and FDH on 2-AET^+^.

Comparing the two H-bonding extremes, the H-bond-diminished 2-TMAET^+^ displayed simultaneous enzyme desorption of (14 ± 1.3)% ((0.45 ± 0.04) pmol cm^–2^) of adsorbed H_2_ase with a current decay of (86 ± 9)% in the first 20 min, indicating that the potential-induced enzyme desorption from the electrode is only possible in the absence of H-bonding (Figure 6a). The same trend was observed for FDH, where negligible enzyme desorption was observed with a current decay of (75 ± 11)% for formate oxidation at +0.4 V vs RHE on 2-AET^+^. Significant FDH desorption of (26 ± 5)% ((0.76 ± 0.16) pmol cm^–2^) was observed with a current decay of (96 ± 6)% for FDH on 2-TMAET^+^ for formate oxidation at +0.4 V vs RHE after 1 h (Figure 6b).

Thus, we provide evidence that H-bonding stabilizes bioelectrocatalysis at the enzyme–electrode interface for both oxidative and reductive reactions.

## Conclusions

We have confirmed the importance of electrostatic interactions for enzyme orientation and activity but also provide strong support for the presence and role of H-bonding in promoting the stability of two model redox enzymes at the enzyme–electrode interface. Both CA and E-QCM confirmed the presence of H-bonding as a dominant non-covalent interaction that, when removed, resulted in the desorption of H_2_ase and FDH films from the electrode due to electrostatic interactions alone being insufficient at preventing desorptive activity loss. We find that resolving other non-covalent interactions outside of electrostatics is critical for a full understanding of the enzyme–electrode interface. Furthermore, a distinction between the total amount of enzymes loaded and the amount of electroactive enzymes wired to the electrode was elucidated by E-QCM. When factored into the calculation of the TOF, it was observed that the intrinsic activity rate for any enzyme directly wired to the electrode is unaffected by the charge of the electrode; however, it remains significantly lower than the solution activity of the enzyme. By understanding the parallels between the enzyme–redox partner interactions in vivo and the enzyme–electrode interface, we have resolved the surface conditions that lead to either desorptive or non-desorptive processes for activity degradation. The understanding of the importance of H-bonding for enzyme stability can help tune the rational design of molecular surfaces to enhance bioelectrocatalytic performances. This underlines the importance of characterizing the presence and function of interactions at the enzyme–electrode interface for future improvements in bioelectrode stability and activity that can help enzymes immobilized on electrodes achieve the exceptionally high rates seen in solution assays that make them such desirable catalytic systems.

## Experimental Section

### Materials

The following chemicals and materials were obtained from commercial suppliers and used without further purification unless otherwise stated: ethanol (VWR Chemicals), hydrogen peroxide (H_2_O_2_, Sigma Aldrich, 33%), sulfuric acid (H_2_SO_4_, Sigma Aldrich, 99%), hydrochloric acid (HCl, Sigma Aldrich, 37%), methyl viologen dichloride hydrate (MV^2+^, Sigma Aldrich, 98%), benzyl viologen dichloride hydrate (BV^2+^, Sigma Aldrich, 98%), Parafilm M (Sigma Aldrich), potassium chloride (KCl, Fisher Chemical), rubber septa (Subaseal), 2-(*N*-morpholino)ethanesulfonic sodium salt (MES, Sigma Aldrich), 4-(2-hydroxyethyl)-1-piperazineethanesulfonic sodium salt (HEPES, Sigma Aldrich), sodium hydroxide (NaOH, Sigma Aldrich, ≥97%), 2-aminoethanethiol hydrochloride (2-AET, Sigma Aldrich, 98%), 2-(bromoethyl)-triethylammonium bromide (Sigma Aldrich, 98%), potassium thioacetate (Sigma Aldrich, ≥ 99%), 3-mercaptopropionic acid (3-MPA, Sigma Aldrich, ≥99%), 2-(dimethyl)aminoethanethiol hydrochloride (2-DMAET, Sigma Aldrich, 95%), 2-mercaptoethanol (2-ME, 99%, Sigma Aldrich), gold(III) chloride trihydrate (HAuCl_4_, Sigma Aldrich, ≥99.9%), sodium borohydride (NaBH_4_, Sigma Aldrich, ≥98.0%), dl-dithiothreitol (DTT, Fisher, ≥98.0%), and sodium formate (Sigma Aldrich, ≥99.0%). Buffer solutions were prepared using water from a Simplicity UV MilliQ system (18.2 MΩ cm at 25 °C) and consisted of MES (50 mM) and KCl (50 mM) or HEPES (50 mM), KCl (50 mM), and sodium formate (20 mM). Gases (CO_2_, N_2_, N_2_ with 2% CH_4_ and H_2_) were supplied by BOC.

The following compounds were synthesized as reported previously: 2-(Trimethylammonium)ethyl thiol (2-TMAET) was synthesized based on a published method.^80^ [NiFeSe]-H_2_ase and [W]-FDH from *D. vulgaris* Hildenborough were expressed, purified, and characterized according to a published method.^27,81^ All purification steps were performed under aerobic conditions at 4 °C. H_2_ase stock solutions (10 μM) with an activity of 5201 ± 293 s^–1^ for H_2_ production were stored in a buffer solution (20 mM Tris–HCl, pH 7.6) at −40 °C under a N_2_ atmosphere. FDH stock solutions (40 μM) with an activity of 1100 s^–1^ for formate oxidation and 320 s^–1^ for CO_2_ reduction were stored in a buffer solution (20 mM Tris–HCl, 10% glycerol, 10 mM NaNO_3_, pH 7.6) at −40 °C under a N_2_ atmosphere.

All measurements with H_2_ase and FDH were carried out in an anaerobic glovebox (MBraun, N_2_ atmosphere, <0.1 ppm O_2_). The potentials for the electrostatic surface contours of enzymes were calculated with the APBS Electrostatics plugin [https://server.poissonboltzmann.org/pdb2pqr] with a correction for charges of the FeS clusters, selenocysteine, nickel, and tungsten in the active site.^82^ PyMOL (version 2.3.4, Schrodinger, LLC) was used for enzyme visualization.

### Physical Methods

^1^H and ^13^C NMR spectra were recorded on a Bruker DPX-400 MHz or a Bruker 500 MHz DCH cryoprobe spectrometer at room temperature. Chemical shifts are given in ppm and coupling constants in Hz. Chemical shifts for ^1^H NMR spectra are referenced relative to residual protons in the deuterated solvent (D_2_O: ^1^H = 4.8 ppm, methanol-d_4_: ^13^C = 49.1 ppm). High resolution-mass spectra (MS) were recorded using a ThermoScientific Orbitrap Classic mass spectrometer.

### Gold Substrate Preparation

Two independent gold disk electrodes of 2 mm diameter (Pine Instruments) were cleaned by immersion in piranha solution (3:1 concentrated H_2_SO_4_/33% H_2_O_2_) for 5 min (*Caution! Piranha solution is very corrosive and may explode if contained in a closed vessel*), then were gently rinsed with Milli-Q water, polished with 0.05 μm alumina (Buehler), and were ultrasonicated in H_2_O followed by EtOH for 2 min. Finally, the electrodes were electrochemically cleaned by repetitive cycling between −0.3 and +1.2 V (vs Ag|AgCl (saturated KCl) in 0.05 M H_2_SO_4_ at a scan rate of 50 mV s^–1^ under N_2_ until a stable voltammogram was observed (around 15 cycles). The amount of charge under the gold oxide reduction peak at +0.9 V vs Ag|AgCl was used by integrating the peak to yield the real electroactive surface area by taking into account the theoretical charge of 390 ± 10 μC cm^–2^ for the reduction of a gold oxide monolayer for the two independent electrodes.^83^ The electrodes were found to have electroactive surface areas (*A*_electroactive_) of (0.165 ± 0.1) and (0.162 ± 0.05) cm^2^. The relevant self-assembled monolayers (SAMs) were formed by immersing the Au substrates in a 10 mM aqueous solution of the relevant thiol overnight.

### Synthesis of 2-(Trimethylammonium)ethyl Thiol (2-TMAET^+^)

The synthesis was performed according to a modified literature procedure.^84,85^ In brief, 2-(bromoethyl)-triethylammonium bromide (5.0 g, 20.2 mmol) was dissolved in 25 mL distilled water. Potassium thioacetate (3.01 g, 26.3 mmol) was added, and the stirred solution was heated to 60 °C. After 16 h, the reaction mixture was concentrated under reduced pressure. The product was extracted by stirring the solid in 100 mL (MeOH/CH_2_Cl_2_, 1:1) at room temperature for 30 min. KBr was removed by filtration over celite. The filtrate was concentrated under reduced pressure, and the extraction/filtration was repeated twice to ensure the removal of all KBr. The product was collected as a red-white solid (2.77 g, 56%), of which 1.5 g was added to hydrochloric acid (HCl, 1 M, 7.5 mL). The reaction mixture was refluxed at 110 °C for 16 h under an inert gas atmosphere. The solvent and volatile by-products were removed in vacuo to yield a white-yellow solid. The product was further purified by dissolution in 0.5 mL MeOH while stirring and continuously heating. At 50 °C, 0.2 mL MeOH was added. At 70 °C, all compound was fully dissolved. The solution was cooled slowly to room temperature and further down to 0 °C with an ice bath. After the product precipitated, the supernatant was removed. To obtain a very pure product, the recrystallization process was repeated while sacrificing the yield. The product was collected as a white hygroscopic solid and dried in vacuo (39 mg, 4%). ^1^H NMR (D_2_O, 400 MHz): δ = 2.95 (2H, CH_2_), 3.15 (9H, NMe_3_), 3.55 (2H, CH_2_). ^13^C NMR (methanol-d4, 101 MHz) δ = 69.56, 53.64, 17.75. MS *m*/*z*: MS calculated for C_5_H_14_NS^+^ 120.08, found 120.12.

### Synthesis of 2-Aminoethanethiol-Capped Gold Nanoparticles

2-Aminoethanethiol-capped gold nanoparticles (2-AET|AuNP) were synthesized using a previously reported method.^86^ In brief, 2-AET (400 μL, 213 mM) was added to HAuCl_4_ (40 mL, 1.42 mM) and was gently stirred for 20 min at room temperature. NaBH_4_ (10 μL, 10 mM) was quickly added, and the mixture was stirred vigorously in the dark at room temperature for 10 min to yield a wine-red solution of 2-AET-AuNPs, roughly 40–50 nm in diameter as determined by dynamic light scattering.

### Preparation of H_2_ase-Modified Electrodes

Enzyme-modified electrodes were prepared in an anaerobic glovebox (MBraun, N_2_ atmosphere, <0.1 ppm O_2_). *Dv*H-H_2_ase (1 μL, 10 μM) was diluted in 4 μL MES (50 mM) with KCl (50 mM) at pH 6 and dropcast onto SAM-modified Au electrodes. The resulting H_2_ase|SAM|Au electrode was left to dry for 15 min and then gently rinsed with buffer to remove any loosely physisorbed enzyme.

### Preparation of FDH-Modified Electrodes

Enzyme-modified electrodes were prepared in an anaerobic glovebox (MBraun, N_2_ atmosphere, <0.1 ppm O_2_). *Dv*H-FDH (1 μL, 40 μM) was mixed in a 1:1 v:v ratio with DTT (50 mM, 1 μL) in MES/KCl at pH 6 for 15 min. The resulting mixture was then diluted in 3 μL MES buffer (50 mM) with KCl (50 mM) at pH 6 and dropcast onto SAM-modified Au electrodes. The resulting FDH|SAM|Au electrode was left to dry for 15 min and then gently rinsed with the buffer to remove any loosely physisorbed enzyme and excess DTT.

### Protein Film Voltammetry

A gas-tight two compartment cell with a Nafion membrane separating the compartments was equipped with a three-electrode setup, consisting of a Ag|AgCl (saturated KCl) reference electrode (BASi). Unless otherwise stated, all potentials are quoted with respect to the reversible hydrogen electrode (RHE) using the conversion *E*_RHE_ = *E*_Ag|AgCl_ + 0.197 + (0.059 × pH) V (25 °C) alongside a Pt wire counter electrode and a H_2_ase|SAM|Au or FDH|SAM|Au rotating disk working electrode (RDE). An electrolyte solution containing MES (50 mM) and KCl (50 mM) at pH 6 for H_2_ase or HEPES (50 mM), KCl (50 mM), and formate (20 mM) at pH 8 for FDH was prepared by dissolving the relevant free acids, their sodium salts, and KCl in ultrapure H_2_O. The electrochemical cell was filled with 8 mL of electrolyte, sealed with rubber septa, constantly kept at 25 °C, and purged with the relevant gas if required for 15 min before the start of the measurement. All electrochemical experiments were performed with an Ivium CompactStat potentiostat and a Pine Instruments rotating disk electrode rotator, and voltammograms were recorded with a scan rate of 5 mV s^–1^ at a rotation speed (ω) of 2000 rpm. Error bars are ± sample standard deviation estimated from at least three experiments across the two independent electrodes. All data processing was performed using Python 3.8.2.

### Electrochemical Quartz Crystal Microbalance Analysis

E-QCM experiments were conducted with a Biolin Q-Sense Explorer module and a custom-designed QCM electrochemical cell in an anaerobic glovebox (MBraun, N_2_ atmosphere, <0.1 ppm O_2_). Typically, a gold-coated quartz chip (0.79 cm^2^) was cleaned using the same procedure as for the gold working electrode, followed by a 15 min UV-ozone treatment, after which the Au sensor was immersed in a 10 mM aqueous solution of the relevant thiol overnight and rinsed with ultrapure water (Milli-Q, >18.2 MΩ.cm) prior to use.

Prior to measuring, an enzyme-free MES buffer solution (50 mM) with KCl (50 mM) at pH 6 was cycled through at 0.141 mL min^–1^ for 10 min to generate a stable baseline. Following this, an enzyme-containing buffer solution (16 nM H_2_ase or 66 nM FDH in MES/KCl (50 mM/50 mM)) was injected into the cell. Enzyme adsorption was quantified by monitoring changes in the resonance frequency of the piezoelectric quartz chip. The frequency was related to the mass through eq 1:^87^
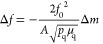
1where *f*_0_ is the resonance frequency of the quartz oscillator, *A* is the piezoelectrically active crystal area, Δ*m* is the change in mass, *p*_q_ is the density of quartz, and μ_q_ is the shear modulus of quartz. To convert the mass adsorbed to quantity of enzyme, an assumption was made that 25% of the adsorbed mass consisted of water molecules bound to the enzyme, which was 91.68 kDa for H_2_ase and 138.3 kDa for FDH in weight.^26,27^

For operando electrochemical analysis, once FDH was fully loaded after 2 h, a 50 mM solution of DTT was injected and flown through the cell for 10 min, after which the flow was stopped, and the solution was kept on the FDH|SAM|Au chip for a further 20 min. Then, the DTT solution was replaced with HEPES/KCl/formate (50 mM/50 mM/20 mM, pH 8), after which electrochemical analysis was carried out. H_2_ase was measured in MES/KCl (50 mM/50 mM, pH 6) as is with no prior activation needed. TOFs were calculated using eqs 2 and 3:
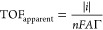
2
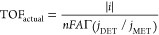
3where *i* is the catalytic current (negative for reductive processes, positive for oxidative processes by convention, calculated from the product of *j*_DET_ and the electrode surface area), *n* is the number of electrons involved in the reaction (2 for the reduction of H^+^ to H_2_, 2 for the oxidation of HCO_2_^–^ to CO_2_), *F* is Faraday’s constant, *A* is the surface area of the electrode (0.79 cm^2^), Γ is the coverage of the enzyme, TOF_apparent_ and TOF_actual_ are the enzyme’s intrinsic rate constant/turnover frequency, and *j*_DET_/*j*_MET_ is the ratio of the direct electron transfer current and the mediated electron transfer current.

For the KCl desorption studies, ionic solutions of KCl in MES (50 mM, pH 6) were prepared and injected into the cell for 30–40 min until no continuous change in frequency was observed. The KCl solution was then replaced by the required buffer solution (MES/KCl (50 mM/50 mM, pH 6) for H_2_ase or HEPES/KCl/formate (50 mM/50 mM/20 mM, pH 8) for FDH for a further 30–40 min until the frequency response stabilized, after which the CVs were recorded. The seventh harmonic (*f*_7_) was used in all data analysis. Errors bars are ± sample standard deviation (s) derived from at least three experiments across at least three independent Au sensors. All data processing was performed using Python 3.8.2.

### Other Instrumentation

The zeta potential and nanoparticle diameter were measured using a Malvern Zetasizer Nano ZS. The sample was dispersed in MES/KCl (pH 6 and 7) and HEPES/KCl (pH 8) solutions (50 mM/50 mM) and allowed to stand prior to measurements in disposable cuvettes (Malvern). Measurements were conducted as three replicates; average results were quoted using the standard deviation as the error.

## Supporting Information Available

Zeta potential measurements of 2-AET|AuNP; protein film voltammograms of H_2_ase and FDH on SAM-modified Au electrodes; protein film voltammograms of FDH on 2-AET^+^ under N_2_ and CO_2_; enzyme-free CVs in the presence and absence of BV^2+^; FDH E-QCM on 2-AET^+^; H_2_ase E-QCM on 3-MPA^–^; E-QCM of sub-monolayer H_2_ase on 2-AET^+^; E-QCM of FDH on 2-TMAET^+^; E-QCM of H_2_ase on propanethiol; E-QCM MET protein film voltammograms of H_2_ase and FDH on 2-AET^+^ and 2-TMAET^+^; table comparing solution assay activities to electrochemical activities for various bioelectrocatalytic systems (PDF)
