# Supplementary material for: Elucidating Film Loss and the Role of Hydrogen Bonding of Adsorbed Redox Enzymes by Electrochemical Quartz Crystal Microbalance Analysis
Source: ACS Catal. 2022 Jan 20;12(3):1886–97. doi: 10.1021/acscatal.1c04317 (PMC9097293; doi:10.1021/acscatal.1c04317)
Supplement: Supplementary file 1 — cs1c04317_si_001.pdf [file cs1c04317_si_001.pdf]

Supporting Information

Elucidating Film Loss and the Role of Hydrogen Bonding of Adsorbed Redox Enzymes by Electrochemical Quartz Crystal Microbalance Analysis

Vivek M. Badiani,[†,‡] Samuel J. Cobb,[†] Andreas Wagner,[†] Ana Rita Oliveira,[§] Sónia Zacarias,[§] Inês A. C. Pereira,[§] and Erwin Reisner[†,*]

† Yusuf Hamied Department of Chemistry, University of Cambridge, Lensfield Road, Cambridge, CB2 1EW, U.K.

‡ Cambridge Graphene Centre, University of Cambridge, Cambridge, CB3 0FA, U.K.

§ Instituto de Tecnologia Química e Biológica António Xavier (ITQB NOVA), Universidade NOVA de Lisboa, Av. da República, 2780-157 Oeiras, Portugal.

[*] To whom correspondence may be addressed. Email: reisner@ch.cam.ac.uk

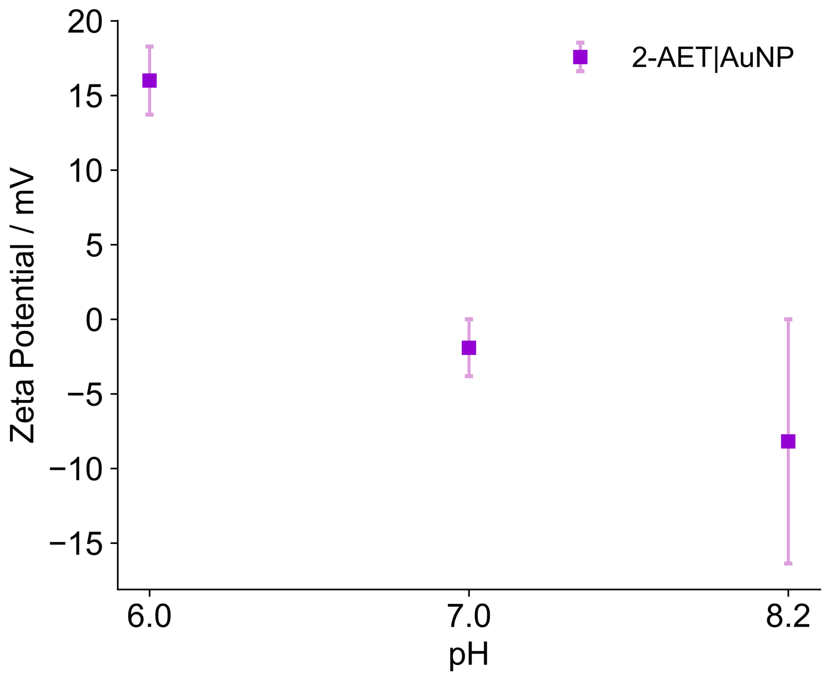

Figure S1. Determination of the electrostatic charge of 2-AET|AuNPs vs pH. The electrostatic charge of the ligand was found to be around +15 mV at pH 6, and drastically decreases to neutral and negative at pH 7.0 and 8.2. This is however, not to be taken as like-for-like with a planar gold surface, as surface topology is expected to shift pK_a values by a few units (*Langmuir* **2002**, *18* (6), 2239–2245).

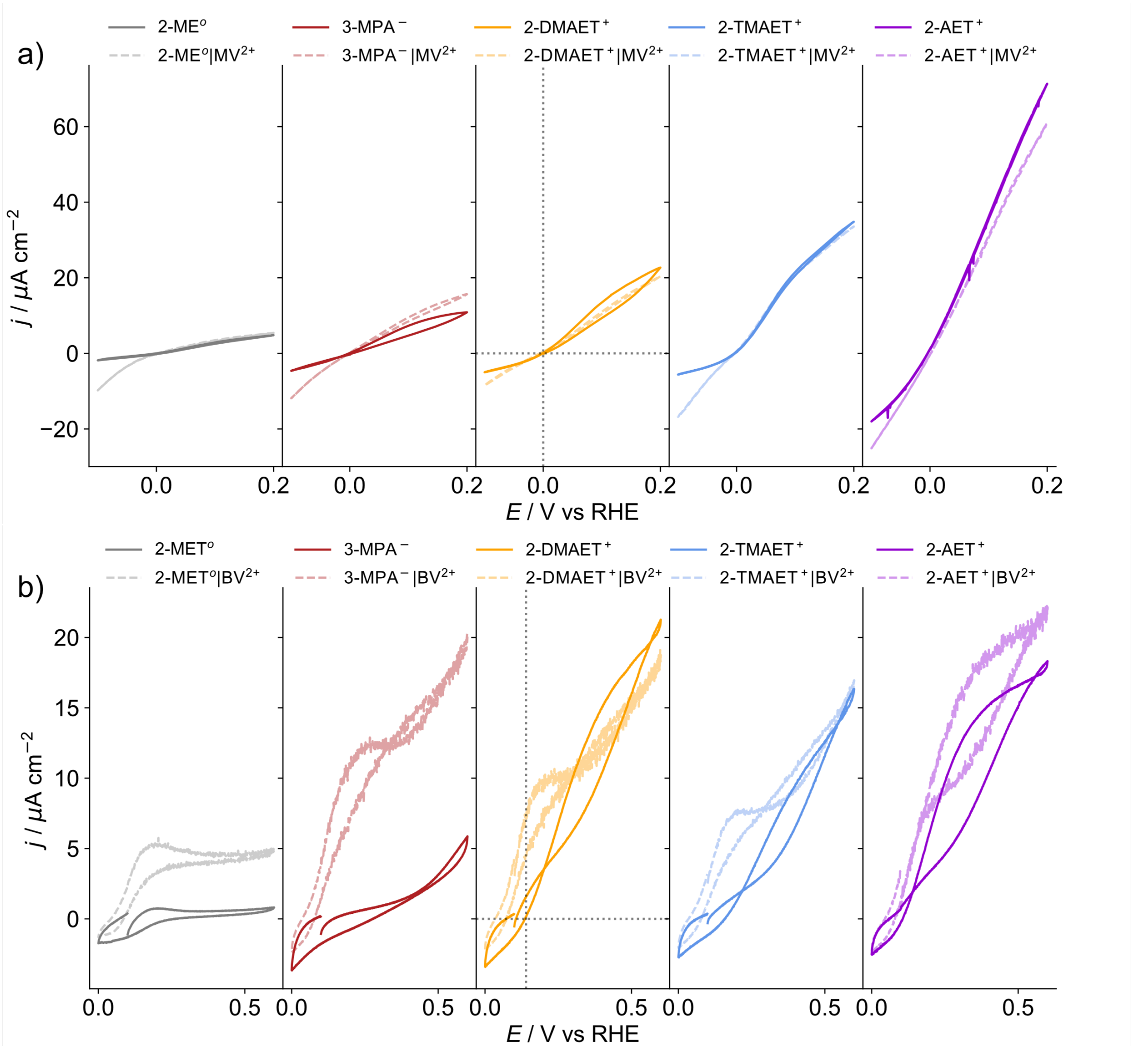

Figure S2. Representative protein film voltammograms of (a) H_2ase and (b) FDH on each SAM-modified Au electrode. The black dotted line on 2-DMAET$^+$ represents the $E^{0'}$ of the H^+/H_2 or CO_2/HCO_2^- couple. The FDH MET protein film voltammograms (dashed lines) are slightly shifted to negative potentials possibly due to the enhanced electron transfer kinetics of the enzyme during MET. Conditions: MES/KCl (50 mM/50 mM, pH 6) 1 atm H_2 for H_2ase (10 pmol), HEPES/KCl/formate (50 mM/50 mM/20 mM, pH 8) for FDH (40 pmol) activated by incubation with 1,4-dithiothreitol (DTT, 50 mM). $\nu = 5$ mV s^{-1}, $\omega = 2000$ rpm, 25°C.

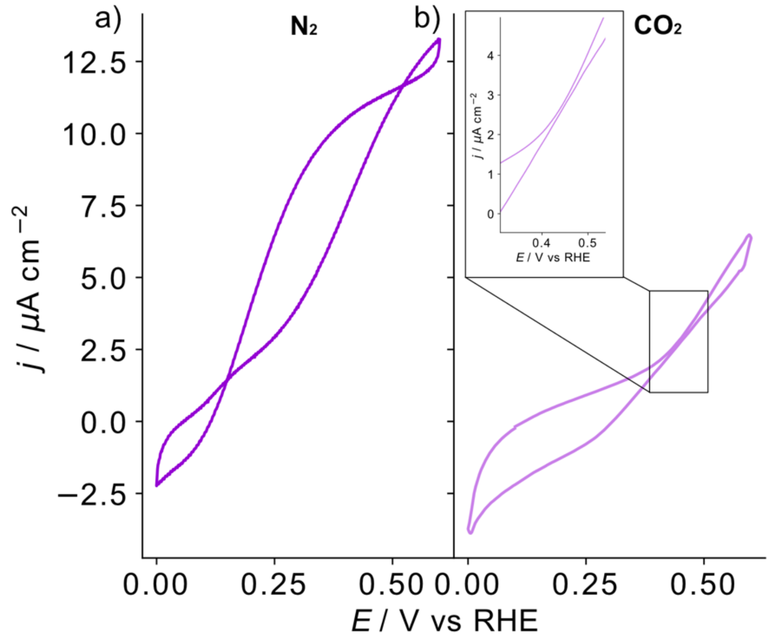

Figure S3. Representative protein film voltammograms of FDH on a 2-AET$^+$|Au electrode under (a) N_2 and (b) CO_2. Conditions: HEPES/KCl/formate (50 mM/50 mM/20 mM, pH 8), 1 atm N_2 or 1 atm CO_2, FDH (40 pmol) activated by incubation with 1,4-dithiothreitol (DTT, 50 mM). $\nu = 5$ mV s^{-1}, $\omega = 2000$ rpm, 25°C.

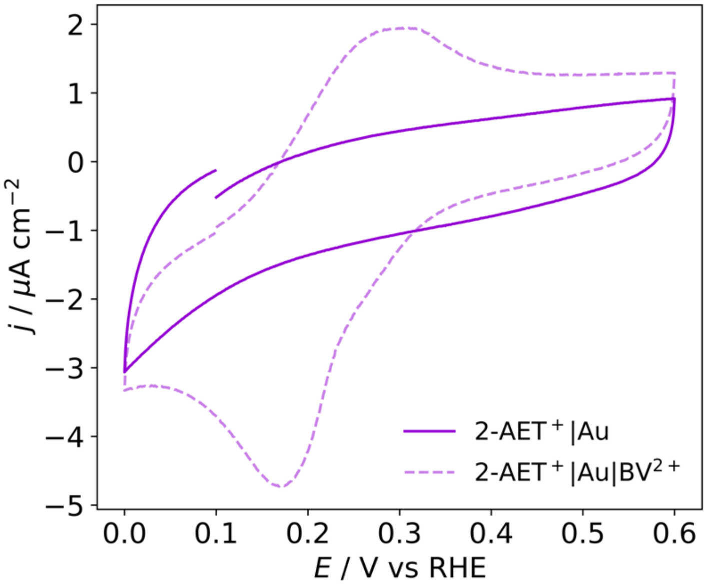

Figure S4. A cyclic voltammogram of an enzyme-free 2-AET$^+$-modified Au electrode both in the absence (solid line) and presence (dashed line) of 250 μM BV^{2+}. Conditions: HEPES/KCl/formate (50 mM/50 mM/20 mM, pH 8), ν = 5 mV s^{-1}, ω = 2000 rpm, 25°C.

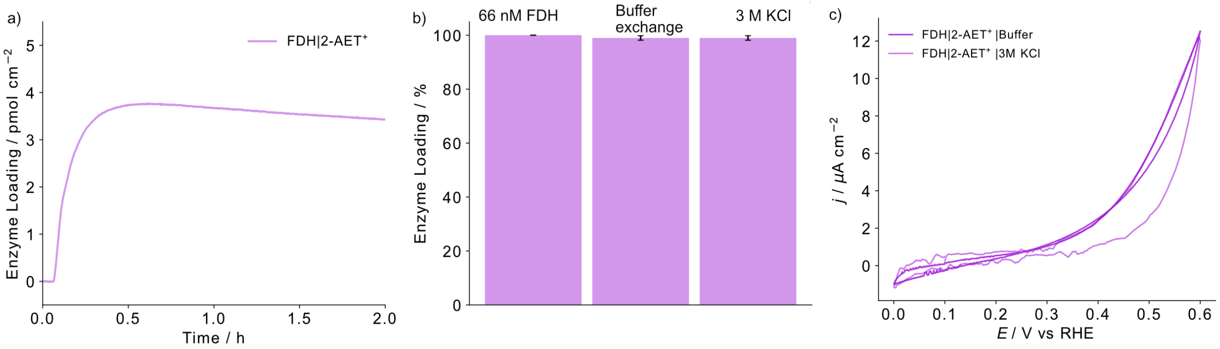

Figure S5. (a) QCM profile for the immobilization of FDH on a 2-AET-modified Au sensor. (b) Desorption profiles of FDH on the SAM-modified Au sensor after a buffer exchange followed by exposure to 3 M KCl. (c) PFV responses of FDH on the SAM-modified sensor after buffer exchange and 3 M KCl. Loading of FDH in MES/KCl (50 mM/50 mM, pH 6), FDH (66 nM), PFV of FDH in HEPES/KCl/formate (50 mM/50 mM/20 mM, pH 8), DTT (50 mM), flow rate = 0.141 mL min^{-1}, 25°C.

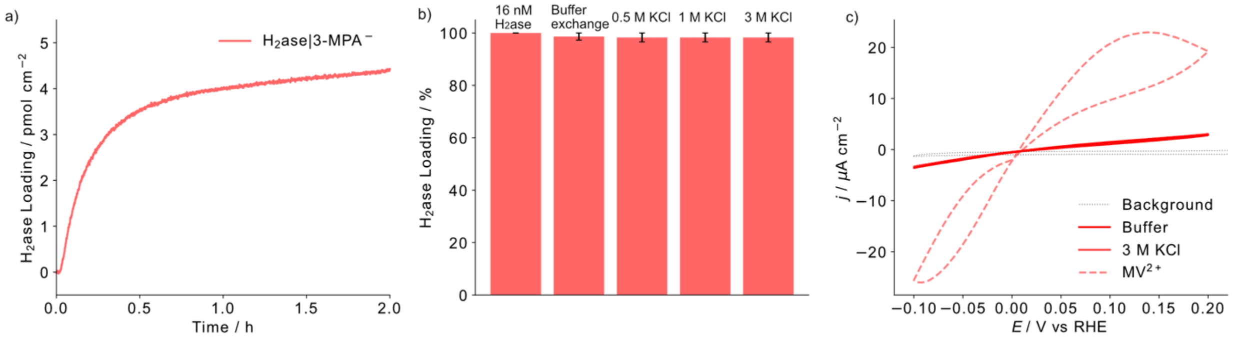

Figure S6. E-QCM study of H_2ase on (a) 3-MPA$^-$ (b) desorption studies of the H_2ase|3-MPA$^-$|Au QCM chip and (c) the PFV responses showing the remaining electrochemical activity of H_2ase after the buffer and 3 M KCl washing step and after the addition of 250 µM MV^{2+}. MES/KCl (50 mM/50 mM, pH 6), 1 atm H_2, 25 °C.

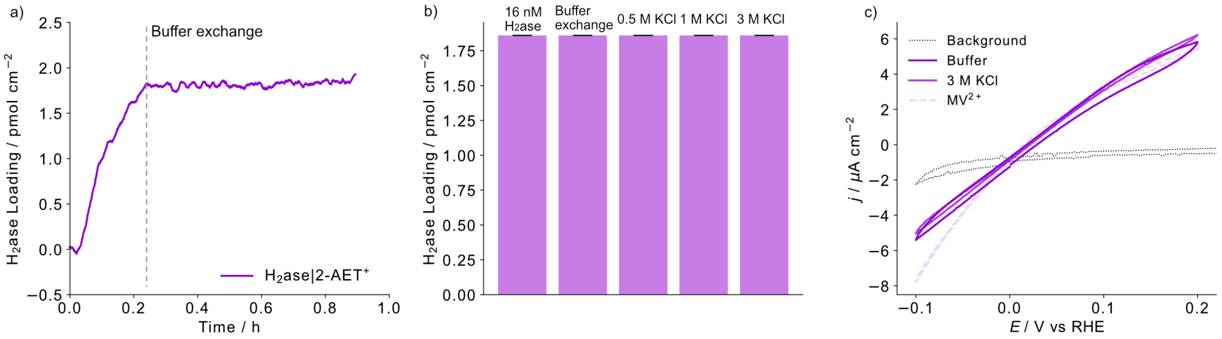

Figure S7. E-QCM study of half loading of H_2ase on (a) 2-AET$^+$, where the H_2ase solution is switched to buffer only after 20 mins to prevent electrode saturation and the loading experiment stopped after 1 h, (b) desorption studies of the half-loaded 2-AET$^+$|Au QCM chip and (c) the PFV responses showing the remaining electrochemical activity of H_2ase after the buffer and 3 M KCl washing step and after the addition of 250 µM MV^{2+}. MES/KCl (50 mM/50 mM, pH 6), 1 atm H_2, 25 °C.

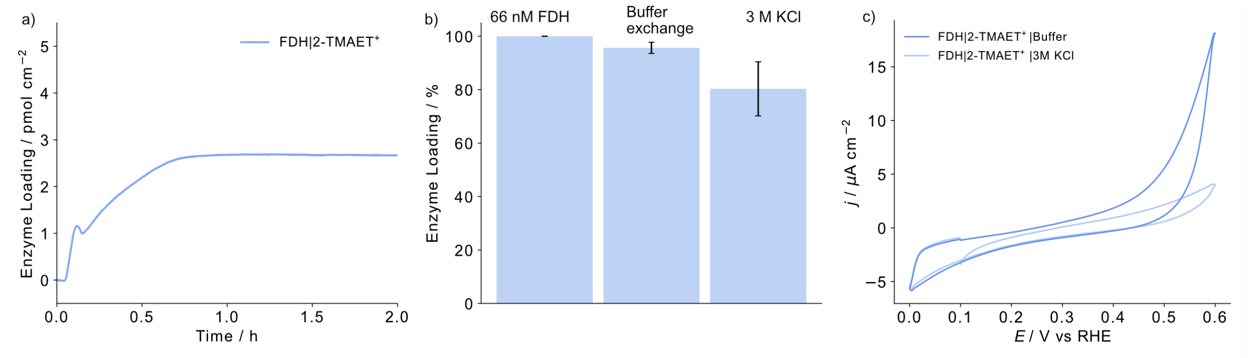

Figure S8. (a) QCM profile for the immobilization of FDH on a 2-TMAET$^+$-modified Au sensor. (b) Desorption profiles of FDH on the SAM-modified Au sensor after a buffer exchange followed by exposure to 3 M KCl. (c) PFV responses of FDH on the SAM-modified sensor after buffer exchange and 3 M KCl. Loading of FDH in MES/KCl (50 mM/50 mM, pH 6), FDH (66 nM), PFV of FDH in HEPES/KCl/formate (50 mM/50 mM/20 mM, pH 8), DTT (50 mM), flow rate = 0.141 mL min^{-1}, 25 °C.

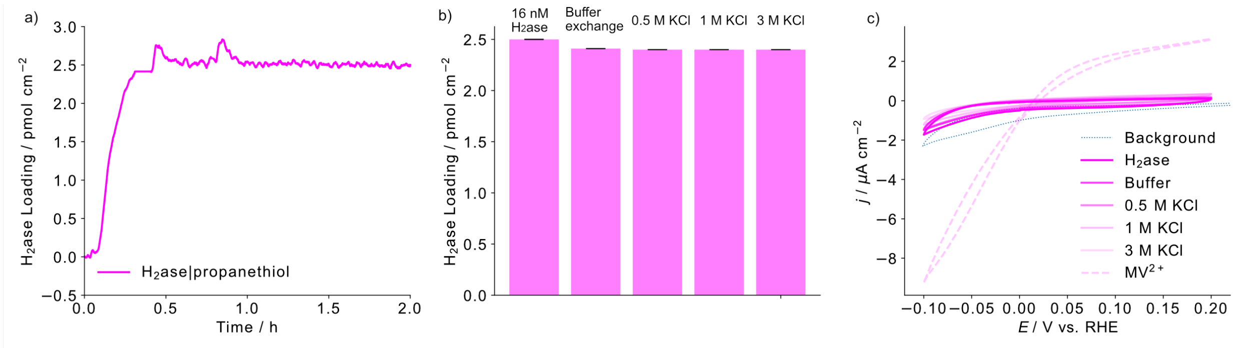

Figure S9. E-QCM study of H_2ase on propanethiol-modified Au where (a) shows the loading plateauing at ca. 2.5 pmol cm^{-2}, (b) shows the desorption studies of H_2ase loaded on propanethiol|Au, with no desorption at 3 M KCl (c) the PFVs showing the low DET electrochemical activity of H_2ase after each KCl washing step, followed by the MET (dashed line) after addition of MV^{2+} (250μM) into the flowing electrolyte to confirm the presence of H_2ase on the electrode. MES/KCl (50 mM/50 mM, pH 6), 1 atm H_2, 25 °C.

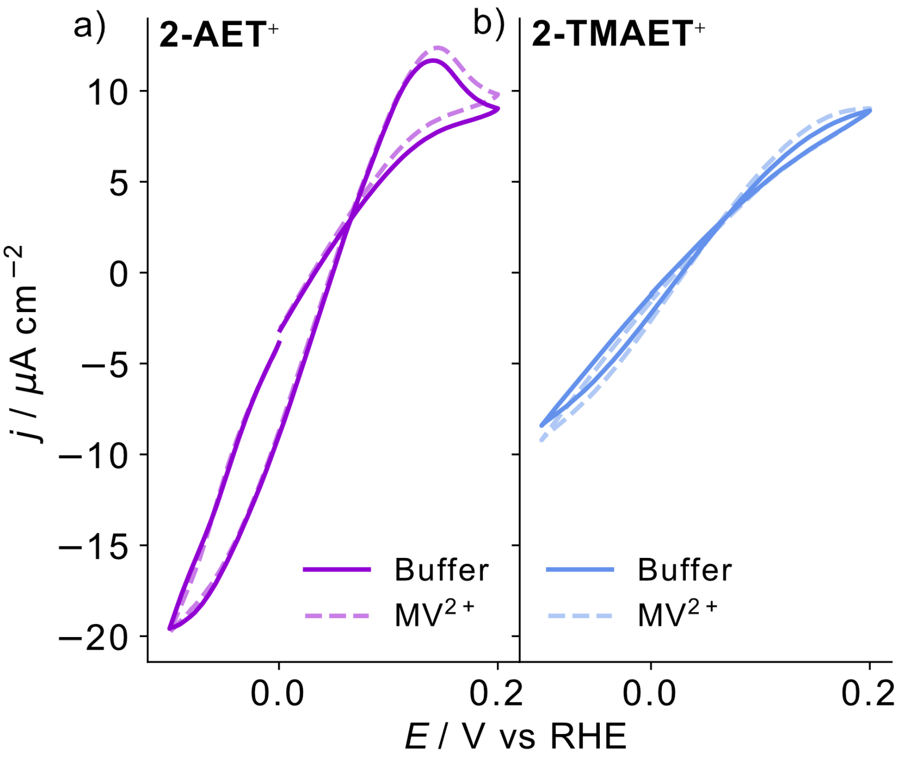

Figure S10. E-QCM PFV responses of H_2ase loaded on (a) 2-AET$^+$-modified sensors and (b) 2-TMAET$^+$-modified sensors before and after the injection of MV^{2+} (250 μM). Conditions: MES/KCl (50 mM/ 50 mM, pH 6), 1 atm H_2, flow rate = 0.141 mL min^{-1}, 25 °C.

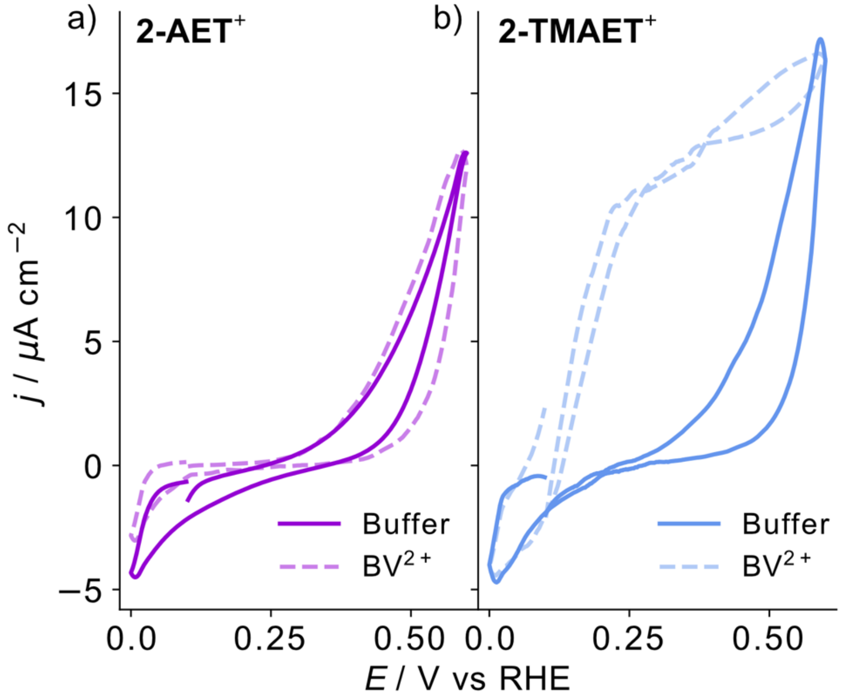

Figure S11. E-QCM PFV responses of FDH loaded on (a) 2-AET$^+$-modified sensors and (b) 2-TMAET$^+$-modified sensors before and after the injection of BV^{2+} (250 µM). The increase in MET for 2-TMAET$^+$ at low overpotentials indicates that electron transfer is initially not as optimal as 2-AET$^+$, however, at high overpotentials j_{DET}/j_{MET} = 1, indicating possible reorientation at high oxidative overpotentials. Conditions: HEPES/KCl/formate (50 mM/ 50 mM, pH 8), 1 atm N_2, flow rate = 0.141 mL min^{-1}, 25 °C.

Table S1. Comparisons of solution assay TOFs and electrocatalytic TOFs for redox enzymes immobilized on different electrodes.

Enzyme[a]	Electrode[b]	Loading (pmol cm^{-2})[c]	Solution TOF (s^{-1})[d, e]	E_{app} (V vs RHE)[f]	Electrode TOF (s^{-1})[g]	Reference
W-FDH (*Sf*)	Pyrolytic graphite edge	3.5	3,380 s^{-1} (+),<br>282 s^{-1} (–)	–0.45	112 s^{-1} (DET, –)	17
W-FDH (*Dv*H)	SAM|Au	8.6	1144 s^{-1} (+)	+0.27	20.5 s^{-1} (DET, +)	23
W-FDH (*Dv*H)	Functionalised graphite	106	1144 s^{-1} (+),<br>236 s^{-1} (–)	+0.27 (+)<br>–0.25 (–)	10.2 s^{-1} (DET, +)<br>3.5 s^{-1} (DET, –)<br>8.6 s^{-1} (MET, –)	23
W-FDH (*Dv*H)	Gas diffusion electrode and redox polymer	650	940 (+),<br>240 s^{-1} (–)	–0.24 (–)	1.7 (DET, –)<br>2.8 (MET, –)	77
W-FDH (*Dv*H)	mesoITO	170	1100 s^{-1} (+),<br>320 s^{-1} (–)	–0.22 (–)	3 s^{-1} (DET, –)	54
W-FDH (*Dv*H)	IO-ITO	104	1100 s^{-1} (+),<br>320 s^{-1} (–)	–0.53 (–)	42 s^{-1} (–)	79
[NiFeSe]-H_2ase (*Dv*H)	IO-ITO	50	8272 s^{-1} (–)	–0.50 (–)	95 s^{-1} (–)	9

[NiFeSe]-H_2ase (*Dmb*)	IO-ITO	80	8700 s^{-1} (+), 2933 s^{-1} (−)	−0.25 (−)	25 s^{-1} (−)	37
BOD (*Mv*)	SAM|Au	4.2	115 s^{-1} (−)	+0.55 (−)	35 s^{-1} (−)	25, 72

[a]*Sf*: *Syntrophobacter fumaroxidans*. *Dv*H: *Desulfovibrio vulgaris* Hildenborough. *Dmb*: *Desulfomicrobium baculatum*. *Mv*: *Myrothecium verrucaria*.

[b]meso: mesoporous. IO: Inverse opal. SAM: Self-assembled monolayer.

[c]Enzyme loading measured by QCM, surface plasmon resonance or estimated from total amount of enzyme drop-cast on the electrode.

[d]The solution TOFs refer to the activity of the enzymes as measured by solution assays. For BOD, the activity is given in units mg^{-1} (1U mg^{-1} = 1 μmol min^{-1} mg^{-1}, ChemPhysChem **2013**, 14, 2097 – 2100), which is converted to s^{-1} by using the enzyme molecular mass of 244.45 kDa.

[e](+) signifies the activity values for the relevant oxidation reaction (formate oxidation or H_2 oxidation). (−) signifies the activity values for the relevant reduction reaction (CO_2 reduction, H^+ reduction).

[f]E_{app} is taken either from chronoamperometry or from a set potential at which a current was used to measure the TOF.

[g]Electrode TOF is calculated from either the current and loading using eq 2 in the Experimental Section or by product quantification.

End of Supporting Information
